# Calciphylaxis Mimicking Penile Gangrene: A Case Report

**DOI:** 10.1100/tsw.2009.150

**Published:** 2009-12-16

**Authors:** Tanweer Ahmed Naweed Bhatty, Kamran Riaz

**Affiliations:** Department of Surgical Specialities, King Fahad Medical City, Riyadh, Saudi Arabia

**Keywords:** calciphylaxis, penis, gangrene

## Abstract

Calciphylaxis is a rare, but life-threatening, disease, mostly seen in patients with renal failure, especially those undergoing dialysis. It is characterized by violaceous tender areas of cutaneous plaques, necrosis, and eschar formation, mostly involving toes and fingers, but rarely the penis. Peripheral pulses are mostly preserved. The parathyroid hormone (PTH) level is elevated, along with raised calcium phosphorus product. There is radiological evidence of blood vessel and soft tissue calcification. Predisposing factors are obesity and diabetes. It is rarely encountered by a urologist and closely resembles penile gangrene.

## CASE REPORT

A 39-year-old male was referred to Urology for penile gangrene. The patient was on hemodialysis via femoral catheter after failed arteriovenous fistula surgery, since 13 years. He had a failed renal transplant 8 years ago. He was oliguric and would hardly pass any urine for days. He was on Warfarin for rheumatic valvular heart disease with right atrial mass ? myxoma ? thrombus and cardiac pacemaker for complete heart block. Examination revealed nontender, blackish discoloration of the glans and adjacent shaft of the penis, resembling penile gangrene, with minimal purulent discharge (Fig. 1). He also had tender plaques with eschar formation involving his middle finger (Fig. 2). He had a raised parathyroid hormone (PTH) level of 344.7 pmol/L (normal value: 1.6–6.9 pmol/L), along with a raised serum phosphate level of 2.29 mmol/L (normal value: 0.81–1.58 mmol/L) and normal serum calcium levels. Plain radiology of the pelvis to check the femoral dialysis catheter position was reported as “calcification of ejaculatory duct and calciphylaxis” (Fig. 3). Skin biopsy is diagnostic, but patient did not consent to it. It was advised to keep the involved area clean and dry after twice daily saline wash. Antibiotics were given according to culture and sensitivity of the wound swab, which grew *Pseudomonas* initially and later *E. coli*. Nephrology management included hemodialysis four times per week, phosphate binder Sevelamer hydrochloride, and Cinacalcet for hyperparathyroidism. The penile lesion improved remarkably by 4 weeks (Fig. 4) and by 16 weeks, both penile and finger lesions almost became normal (Figs. 5 and 6).

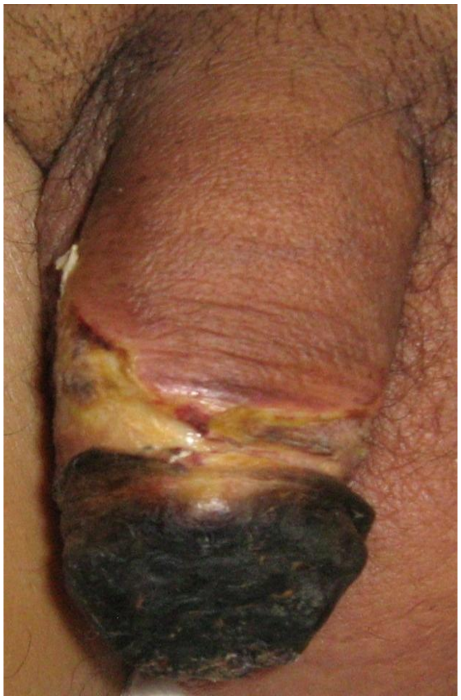

**FIGURE 1.** Calciphylaxis mimicking penile gangrene.

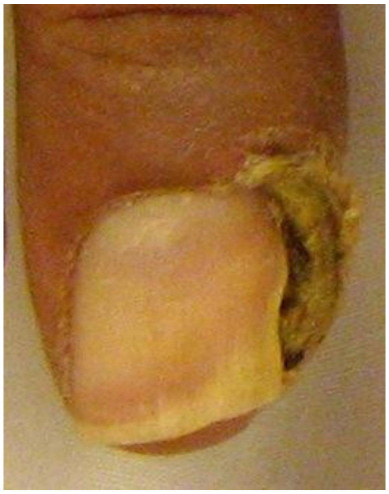

**FIGURE 2**. Calciphylaxis involving middle finger.

## DISCUSSION

Calciphylaxis was first reported in 1898 by Bryant and White, who described an association between cutaneous gangrene and vascular calcification[1]. Calciphylaxis typically involves the lower extremities and is associated with renal failure on dialysis, elevated PTH, with dysregulation of calcium and phosphorus metabolism, frequently with fatal outcome[2]. Its mean age of presentation is 48 years (range: 6 months to 83 years), with small- and medium-sized blood vessel calcification as the most specific radiological finding[2]. Mammography is superior to plain radiology or CT scan in showing blood vessel calcification[3]. Calciphylaxis has been given different names, including uremic gangrene syndrome, and

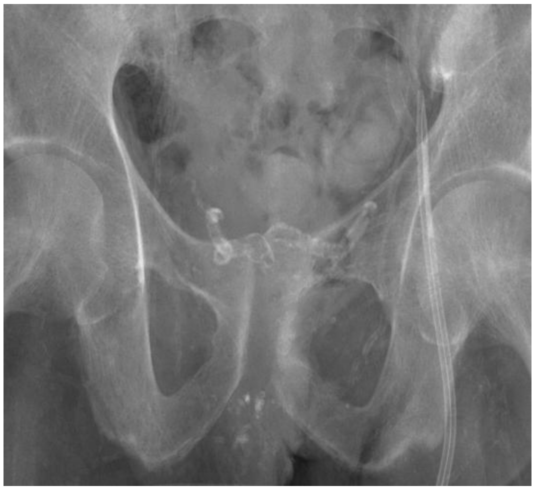

**FIGURE 3.** Plain film showing calcification of ejaculatory duct and calciphylaxis.

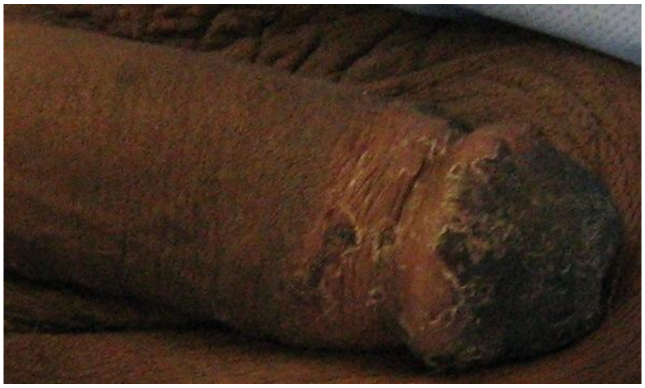

**FIGURE 4.** Result after 4 weeks of conservative management.

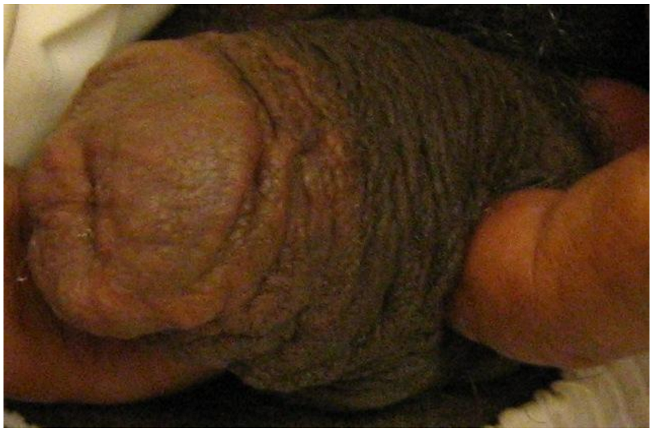

**FIGURE 5**. Result after 16 weeks of conservative management.

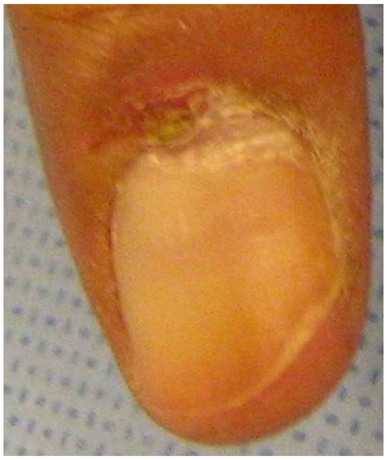

**FIGURE 6**. Result after 16 weeks of conservative management.

develops in 1–4% of patients with end-stage renal disease, but involvement of the penis is rare[4,5]. Calciphylaxis has been reported in the absence of renal failure[6]. Some studies suggest better prognosis in distal involvement of fingers, toes, or penis compared with proximal involvement of thighs, buttocks, and trunk[7]. Lesions present with painful violaceous papules, which subsequently ulcerate and become necrotic, carrying a high mortality rate of 60–69% from sepsis[8]. Medical management includes adjusting calcium and phosphate levels, and secondary hyperparathyroidism, while the role of parathyroidectomy is controversial[9]. Surgical debridement of the leision is also controversial[10].

## CONCLUSIONS

A urologist rarely comes across a penile lesion secondary to calciphylaxis, which so closely resembles penile gangrene. It requires a multidisciplinary approach involving urology and nephrology. Nephrologists need to correct serum calcium-phosphate dysregulation and secondary hyperparathyroidism. We successfully managed the case conservatively and advise the same, with very close observation. If it fails, then surgery is the only option to save the patient’s life.
